# Re-analysis of whole-exome sequencing data uncovers novel diagnostic variants and improves molecular diagnostic yields for sudden death and idiopathic diseases

**DOI:** 10.1186/s13073-019-0702-2

**Published:** 2019-12-17

**Authors:** Elias L. Salfati, Emily G. Spencer, Sarah E. Topol, Evan D. Muse, Manuel Rueda, Jonathan R. Lucas, Glenn N. Wagner, Steven Campman, Eric J. Topol, Ali Torkamani

**Affiliations:** 1 Scripps Research Translational Institute at Scripps Research, La Jolla, CA USA; 2 Division of Cardiology, Scripps Clinic, La Jolla, CA USA; 3 Los Angeles County Department of Medical Examiner-Coroner, Los Angeles, CA USA; 4 San Diego County Medical Examiner’s Office, San Diego, CA USA

**Keywords:** Whole-exome sequencing, Medical genetics, Molecular autopsy, Rare and undiagnosed diseases, Sudden death, Automated periodic re-analysis

## Abstract

**Background:**

Whole-exome sequencing (WES) has become an efficient diagnostic test for patients with likely monogenic conditions such as rare idiopathic diseases or sudden unexplained death. Yet, many cases remain undiagnosed. Here, we report the added diagnostic yield achieved for 101 WES cases re-analyzed 1 to 7 years after initial analysis.

**Methods:**

Of the 101 WES cases, 51 were rare idiopathic disease cases and 50 were postmortem “molecular autopsy” cases of early sudden unexplained death. Variants considered for reporting were prioritized and classified into three groups: (1) diagnostic variants, pathogenic and likely pathogenic variants in genes known to cause the phenotype of interest; (2) possibly diagnostic variants, possibly pathogenic variants in genes known to cause the phenotype of interest or pathogenic variants in genes possibly causing the phenotype of interest; and (3) variants of uncertain diagnostic significance, potentially deleterious variants in genes possibly causing the phenotype of interest.

**Results:**

Initial analysis revealed diagnostic variants in 13 rare disease cases (25.4%) and 5 sudden death cases (10%). Re-analysis resulted in the identification of additional diagnostic variants in 3 rare disease cases (5.9%) and 1 sudden unexplained death case (2%), which increased our molecular diagnostic yield to 31.4% and 12%, respectively.

**Conclusions:**

The basis of new findings ranged from improvement in variant classification tools, updated genetic databases, and updated clinical phenotypes. Our findings highlight the potential for re-analysis to reveal diagnostic variants in cases that remain undiagnosed after initial WES.

## Background

Early sudden unexplained death and rare undiagnosed disorders have major impacts on affected individuals as well as their family members. Three hundred thousand to four hundred thousand people per year in the USA alone die from sudden death-related conditions [1], and rare diseases occur cumulatively at an estimated population frequency of 10% [2]. Both conditions can often be linked to genetic, often monogenic, risk factors. Whole-exome sequencing (WES) is a powerful approach for the identification of these genetic risk factors. However, the genetic and phenotypic heterogeneity of these conditions can make identifying a molecular diagnosis challenging. The diagnostic yield of exome sequencing ranges from 15 to 50% depending upon the stringency of inclusion criteria and phenotype in question [3–6]. Thus, even in cohorts most stringently recruited and most enriched with likely monogenic conditions, significant gaps remain in achieving expected diagnostic yield.

Re-analysis of WES data could improve diagnostic rates in patients without an initial molecular diagnosis; however, the procedures, timing, expected yield, and source of improved diagnostic yield for re-analysis have only recently been evaluated in a limited number of long-running WES programs [7–15]. Therefore, we re-interpreted two WES-based studies performed at The Scripps Research Translational Institute with 101 combined cases initially interpreted between 1 and 7 years ago. These two programs include 51 cases of rare, idiopathic, likely monogenic disorders and 50 cases of early, potentially genetic, sudden unexpected death [16, 17]. We assessed the increase in diagnostic yield after re-analysis and evaluated the factors leading to new reportable findings. Re-analysis resulted in the identification of additional diagnostic variants in 3 rare disease cases (5.9%) and 1 sudden unexplained death case (2%). New findings were determined to be due to either initially incomplete phenotypic information (i.e., affection status of family members) or incomplete or inaccurate annotation information [18]. Newly available clinical information and genetic knowledge as well as improvements to our bioinformatic pipeline substantially increased combined diagnostic yield by 18%, from 17.8 to 21.8%. The absolute diagnostic yield increased from 25.4 to 31.4% for rare disease and 10 to 12% for sudden death.

## Methods

### Study design

Participants were enrolled in two studies from 2011 to 2018; a rare disease study—Idiopathic Diseases of huMan (IDIOM), and a post-mortem genetic testing study in early sudden death—Molecular Autopsy (MA). The inclusion criteria, prospective recruitment strategy, phenotyping, and initial analysis approach for these studies are described in detail elsewhere [16, 17]. In brief, the IDIOM study aims to discover novel gene–disease relationships and provide molecular genetic diagnosis and treatment guidance for individuals with novel diseases using genome sequencing integrated with clinical assessment and multidisciplinary case review, whereas the MA study seeks to incorporate prospective genetic testing into the postmortem examination of cases of sudden unexplained death in the young (< 45 years old). Under these protocols, we recruited 101 analyzable proband participants altogether: 51 proband participants (including 4 singletons) were enrolled in the IDIOM study from 2011 to 2018, while 50 deceased individuals and their living relatives were enrolled in the MA study from 2014 to 2018. The IDIOM study (IRB-11–5723) and the Scripps Molecular Autopsy study (IRB-14-6386) were both approved by the Scripps Institutional Review Board.

### Whole-exome sequencing

Detailed procedures for WES have been described previously [16, 17, 19, 20]. In brief, whole blood samples were preserved using Paxgene DNA tubes (PreAnalytiX, Hombrechtikon, CH), and genomic DNA was extracted using the QIAamp system (Qiagen, Valencia, CA). Enriched exome libraries were captured using a variety of Agilent SureSelect systems according to the manufacturer’s instructions (Agilent, Santa Clara, CA). Final libraries were generated using Illumina TruSeq sample preparation kits and underwent 100 bp paired-end sequencing on a HiSeq 2500 (Illumina, San Diego, CA). Samples were sequenced to a median coverage of 98X in combined studies.

### Variant calling and annotation

The original downstream analysis procedure has been described in detail previously [16]. In brief, alignment and variant calling were performed using BWA-GATK best practices (which changed significantly especially over the duration of the IDIOM protocol) [21]. Annotation and variant prioritization were performed using the SG-ADVISER system.

For our re-analysis, each WES sample was processed using the Genoox platform, which employs Burrows–Wheeler Aligner (version 0.7.16) [22] for the mapping of short-read sequences using *hg19* as reference, Genome Analysis Toolkit (GATK; version 4.0.7.0) [23, 24], and FreeBayes (version 1.1.0) [25] for variant calling of low-frequency SNVs, multiple nucleotide variants (MNVs), and INDELS.

### Variant filtration and prioritization

After annotation, an automated variant filtration pipeline was applied to narrow down the number of candidate diagnostic SNVs and INDELS using the following rules: (1) variants that follow disease segregation in the family—including multiple probands; (2) functional impact-based filtration retaining only variants that are non-synonymous, frameshift, and nonsense, or affect canonical splice-site donor/acceptor sites; and (3) variants with a minor-allele frequency (MAF) < 1% in population-level allele frequency data derived from the Exome Aggregation Consortium (ExAC), 1000 Genomes Project (1000G), Exome Variant Server (ESP), 10,000 UK Genome (UK10K), The Genome Aggregation Database (gnomAD), and internal data from our studies.

### Automated variant classification engine

Further variant prioritization was then performed by combining annotation information into a summary interpretation of variant pathogenicity. For our initial studies, variant interpretation was carried out as described previously and in accordance with the criteria set by the American College of Medical Genetics and Genomics (ACMG)/Association for Molecular Pathology (AMP) guidelines as previously described [26, 27]. In addition, we incorporated the recommendations from the ClinGen Sequence Variant Interpretation (SVI) working group for using ACMG-AMP criteria, regarding the exclusion of the two reputable source criteria pertaining to variant classification PP5 and BP6 due to their questionable validity [28]. For our re-analysis, Genoox (https://www.genoox.com), an artificial intelligence-based variant classification and interpretation engine, was used, which builds disease association and deleteriousness prediction models at the gene and variant level by integrating information from various gene and variant classification sources (e.g., ClinVar, ClinGen, Uniprot, gnomAD, ExAC, Orphanet) [29]. To mitigate limitations in computationally extracting the exact evidence for which the submission is based on (e.g., ClinVar, UniProt, and the literature) since these are currently unstructured, the classification engine applies PP5/BP6 to help prioritize and alert about previously reported variants, or suggest for being clinically relevant. Similarly, based on different features (e.g., number of submitters, dates, type of submitters, number of publications), the strength of the evidence can be estimated. The reported evidence under the PP5/BP6 rules is then manually applied with the relevant rules instead of PP5/BP6, to comply with the new recommendations. Although the actual classification is not affected, it is rather how their evidence is presented. Variants were classified into one of five categories: benign (B), likely benign (LB), variant of uncertain significance (VUS), likely pathogenic (LP), and pathogenic (P). VUS were then further classified by using a combination of in silico prediction tools including (1) missense deleteriousness prediction tools (including REVEL, MetaLR, MT, MA, FATHMM, SIFT, CADD, and POLYPHEN2) [30], (2) splicing defect prediction tools (dbscSNV Ada, Splice AI), (3) conserved region annotation (GERP), and (4) whole-genome functional annotation (GenoCanyon, fitCons, ncER [31]). VUS subclassifications were (1) VUS-PB, if additional evidence was found to support the variant as being Possibly Benign (e.g., non-coding variant not predicted to influence splicing); (2) VUS-U, if there was some evidence for pathogenicity based on variant class but limited additional evidence of deleteriousness (e.g., non-synonymous variant with tolerated and damaging effect according to respective prediction tools); and (3) VUS-PP (possibly pathogenic), if there was strong evidence for pathogenicity based on computational evidence supporting a deleterious effect on the gene or gene product, but not sufficient evidence to meet the likely pathogenic classification according to ACMG-AMP guidelines [27].

### Gene-level evidence

Genes with candidate variants were considered for return if the gene had at least a strong level of evidence as outlined in the ACMG/AMP guidelines for association with a monogenic disease. Variants in genes with moderate evidence were also chosen for return if agreed upon after discussion with the broader research team and physician review panel.

For sudden death cases, to be considered diagnostic, the gene must be present in our curated list of confirmed or probable genes associated with sudden unexplained death (SUD), sudden cardiac death (SCD), and sudden death in epilepsy (SUDEP). Our gene panel was drawn from multiple sources, including Human Gene Mutation Database (HGMD), Online Mendelian Inheritance in Man (OMIM), ClinVar, Uniprot, and a combination of several gene panels associated with sudden cardiac death, sudden death in epilepsy, channelopathies, and genetic connective tissue disorders. The content of our list evolved throughout the study as sources were updated. This list contains a total of 1608 genes, and all have been previously cataloged in The Genetic Testing Registry (GTR) and The Genomics England PanelApp (https://panelapp.genomicsengland.co.uk/panels/) as associated with the following conditions: GTR: arrhythmogenic right ventricular cardiomyopathy, comprehensive cardiology, arrhythmia, cardiac arrhythmia, long QT/Brugada syndrome, inherited cardiovascular diseases and sudden death, cardiomyopathies, comprehensive cardiomyopathy, comprehensive arrhythmia, catecholaminergic polymorphic ventricular tachycardia, cardiac arrhythmia, sudden death syndrome, comprehensive cardiovascular, cardiovascular diseases, familial aneurysm, connective tissue disorders, epilepsy, and seizure. PanelApp: dilated cardiomyopathy—adult and teen, dilated cardiomyopathy and conduction defects, idiopathic ventricular fibrillation, long QT syndrome, sudden death in young people, molecular autopsy, brugada syndrome, mitochondrial disorders, familial hypercholesterolemia, thoracic aortic aneurysm or dissection, epilepsy—early onset or syndromic, and genetic epilepsy syndromes.

### Combined evidence for reporting

The final assessment of pathogenicity was determined by integrating patient assessment, variant evaluation, inheritance, and clinical fit. The following final classifications were used for reporting:

- Category 1. Diagnostic variants (DV): Known pathogenic or likely pathogenic variant(s) either (1) in a known disease gene associated with the reported phenotype provided for the IDIOM proband or (2) in a known gene associated with sudden death for deceased MA individuals. Findings in this category are reported as positive.
- Category 2. Possible diagnostic variants (PDV): Pathogenic variant(s) in known disease genes possibly associated with the reported IDIOM phenotype, or possibly pathogenic variants in genes known to be associated with sudden death in MA. This category also includes single pathogenic or likely pathogenic variants identified in a gene associated with an autosomal recessive disorder consistent or overlapping with the provided IDIOM. Findings in this category are reported as plausible but negative.
- Category 3: Variants of uncertain diagnostic significance (VUDS): Variant(s) predicted to be deleterious in a novel candidate gene not previously implicated in human disease, or with an uncertain pathogenic role, in the presence of additional supporting data. Such data may include animal models, copy number variant data, tolerance of the gene to sequence variation, tissue or developmental timing of expression, or knowledge of the gene function and pathway analysis. Further research is required to evaluate and confirm any of the suggested candidate genes. Findings in this category are reported as negative.
- Category 4 (negative result; negative): No variants in genes associated with the reported phenotype were identified.

Read-level data was visually inspected for variants considered for reporting and validated via Sanger sequencing if determined to be necessary. Amended reports were returned to the referring physician when new diagnostic variants were identified. This new report includes full interpretation of any newly identified variants and updated classifications of previously identified variants where applicable.

## Results

A total of 577 variants were considered for further analysis by our variant annotation and filtering workflows across both IDIOM and MA studies, an average of ~ 5.3 variants per subject (Additional file 1: Table S1 and Table S2). Through the use of a computational phenotype-driven ranking filter, 117 variants were prioritized as likely or previously reported pathogenic and potentially associated with the proband’s phenotype (Additional file 1: Table S3A and Table S3B) and 81 variants were considered damaging but lacked direct evidence for pathogenicity, while a further 379 variants displayed either a lack of relevance of gene to phenotype, or did not match the expected genetic model based on phenotype segregation in the family. From our list of 117 candidate diagnostic variants, 40 were reportable and concordant with the phenotypic descriptions of the probands.

For rare disease, we identified a diagnostic variant in 16 probands from the IDIOM study, corresponding to a diagnostic yield of 31.4%. Three of 16 cases were new findings after re-analysis, corresponding to an increase in diagnostic yield of 23% (from a yield of 25.5 to 31.4%). Of all findings, 50% were de novo mutations and 50% were inherited variants (37.5% recessively inherited from both parents, 6.25% dominantly inherited from an affected parent, 6.25% inherited variation in mitochondrial DNA). An additional 18 IDIOM probands (35.2%) have variants of uncertain diagnostic significance in known disease-associated genes, some of which may become diagnostic in future as further evidence accumulates (Additional file 1: Table S3A and Table S4A).

For sudden death, we identified diagnostic variants in 6 probands, corresponding to a diagnostic yield of 12%. One of 6 cases was a new finding after re-analysis, corresponding to an increase in diagnostic yield of 20% (from a yield of 10% to 12%). Nearly half of all our sudden death cases (42%) had a possible diagnostic variant in suspected/known sudden death-associated genes, yet most lack the evidence required to support definitive claims of pathogenicity for sudden death. An additional 8 MA probands (16%) have variants of uncertain diagnostic significance in suspected/known sudden death-associated genes, of which 3 MA cases had no variant identified in our initial study (Additional file 1: Table S3B and Table S4B).

In total, 4 cases received a revised report with a novel diagnostic variant (Table 1), all 18 prior positive findings were confirmed, and potentially informative variants were identified in 11 (10.7%) cases that previously had no candidate variants for consideration (Additional file 1: Table S4A and Table S4B). Of the new diagnoses, 1 resulted from revised family history, 2 were due to corrected variant misannotation, and 1 was due to corrected gene-disease association (Table 1). Brief clinical descriptions of the new findings and the reason for identification of the new findings are described below:

**Table 1:** Diagnostic variant observed following exome filtering and interpretative assessment after re-analysis

	ID00024	ID00038	ID00048	MA02003
Case description and initial exome analysis				
Primary clinical features at referral	Seizures; decreased white brain matter; neuropathy	Global developmental delay; microcephaly; intellectual disability	Short stature with deformities of lower extremities; muscle tone decreased throughout; spine with mild scoliosis	History of depressive disorder and anxiety; seizures; sudden death
Exome quality metrics	Average coverage: 83.7X	Average coverage: 101.3X	Average coverage: 78X	Average coverage: 118X
	% of covered bases at ≥ 20x: 93.8%	% of covered bases at ≥ 20x: 92.4%	% of covered bases at ≥ 20x: 94%	% of covered bases at ≥ 20x: 95.7%
Initial exome interpretation	No clinically relevant variant (2015)	No clinically relevant variant (2016)	No clinically relevant variant (2017)	No clinically relevant variant (2016)
Exome reanalysis				
Candidate variant (Hg19)	chr1:154,560,601 G > A	chr12:109,921,417 G > T; chr12:109,948,147 A > G	chr17:76,993,476 A > AG; chr17:76991236 C > A	chr12:111,348,980 G > C
HGVS Nomenclature	NM_001111.5 (*ADAR*):c.3019 G > A	NM_130466.4 (*UBE3B*):c.61 G > T; NM_130466.4 (*UBE3B*)c.1742-2 A > G	NM_001159773.2 (*CANT1*):c.228dupC; NM_001159773.2 (*CANT1*)c.699 G > T	NM_000432.3 (*MYL2*):c.403-1 G > C
ACMG/AMP Criteria	PS1- strong; PS3-strong; PM2-moderate	PVS1 (c.1742-2A>G; c.61G>T); PS1- strong (*c.61G>T);* PM2-moderate (c.1742-2A>G; c.61G>T)	PVS1 (c.228dupC); PS3- strong (*c.*228dupC); PM2-moderate (c.228dupC; *c.699 G > T*); PP3-supporting (c.699 G > T); PP2-supporting (*c.699 G > T*)	PVS1; PS3- strong; PM2-moderate
Re-analysis assessment	Pathogenic (DV)	Pathogenic (DV; *c.61G>T*); likely pathogenic (DV; *c.1742-2A>G*)	Pathogenic (DV; c.228dupC); possibly pathogenic (PDV; *c.699 G > T*)	Pathogenic (DV)
Diagnosis (OMIM)	Aicardi-Goutieres Syndrome 6 (615010) [32]	Kaufman Oculocerebrofacial Syndrome (244450) [33]	Multiple epiphyseal dysplasia 7 (617719); Desbuquois dysplasia 1 (251450) [34]	Familial hypertrophic cardiomyopathy 10 (608758) [35]
Prognosis	Heterogeneous disease characterized by cerebral atrophy, leukoencephalopathy, intracranial calcifications, chronic cerebrospinal fluid (CSF) lymphocytosis, increased CSF alpha-interferon. Death often occurs in early childhood.	Unusually small head size (microcephaly), structural abnormalities of the brain. Affected individuals have weak muscle tone (hypotonia) and are delayed in developing motor skills such as walking. Intellectual disability is severe or profound.	Mixture of the features observed in MED and DD. MED is a disorder of cartilage and bone development, primarily affecting the ends of the long bones in the arms and legs. DD is a more severe form of chondrodysplasia overall.	Alteration of cardiac contraction which provoked changes in myofibrillar Ca2+ sensitivity, subsequently it could lead to diastolic dysfunction and sensitivity to dysrhythmias, which at times cause sudden death.

### IDIOM24

IDIOM24, a 12-year-old girl of European ancestry, presented with seizures, spasticity, gastroesophageal reflux, and neuroimaging, showed decreased cerebral white matter. The proband underwent extensive clinical investigation, including electroencephalography, brain magnetic resonance imaging, single-photon emission computed tomography brain scan, EMG/nerve conduction studies, and muscle biopsy, but these workups failed to provide a diagnosis, and numerous therapeutic interventions were tried without lasting benefit.

A dominantly acting known pathogenic variant, *ADAR (p.Gly1007Arg; rs398122822; NM_001111.5)* was automatically removed from consideration during the initial analysis for IDIOM24 due to incomplete phenotypic information regarding the proband’s biological father. The variant was called as shared by the affected proband and presumably unaffected biological father. Automatic identification of the pathogenic variant during re-analysis and re-investigation of family history resulted in the re-identification and prioritization of this pathogenic variant. Somatic mosaicism was confirmed in the biological father, and diagnosis was corroborated by the physician.

### IDIOM38

IDIOM38, a 3-year-old girl of mixed ancestry, presented with global developmental delay, intellectual disability, microcephaly, and malformed right ear. The proband required the placement of a gastrostomy tube (G-tube) and underwent brain MRI. Clinical features were run through the London dysmorphology database, and chromosomal analysis and oligonucleotide SNP array were performed. No conclusive diagnosis could be made.

Compound heterozygous variants, *UBE3B (c.1742-2A>G; c.61G>T; NM_130466.4)*, had been identified as candidates but not prioritized for reporting due to incomplete annotation regarding the relationship between *UBE3B* and disease. Compound heterozygous pathogenic and likely pathogenic variants were identified during re-analysis and prioritized due to phenotype match.

### IDIOM48

IDIOM48, a 4-year-old girl of European ancestry, presented with short stature with deformities of lower extremities, spine with mild scoliosis, ligament laxity, and congenital malformation. The proband underwent spine MRI and karyotyping, but no diagnosis could be established.

Compound heterozygosity of *CANT1 (c.228dupC; c.699G>T; NM_001159773.2)*, was not identified during the initial analysis due to a corrupt pre-annotation database entry resulting in the misannotation of the contributing missense variant as a non-coding variant. Corrected variant annotation resulted in the identification of *CANT1* compound heterozygosity due to the newly identified missense variant occurring in trans to the likely pathogenic frameshift variant. The identification of these compound heterozygous variants in *CANT1* revealed a blended phenotype caused by a pathogenic and possibly pathogenic variations, leading to overlapping clinical features of Multiple Epiphyseal Dysplasia and Desbuquois Dysplasia.

### MA02003

A clinical autopsy of MA02003 documented a well-developed, adequately nourished 21-year-old male with no indication as to the cause of death. The cardiovascular pathology report revealed no significant narrowing by atherosclerosis disease. No anatomic cause of death was identified after autopsy.

A dominantly acting variant, *MYL2 (c.403-1G > C; rs199474813; NM_000432.3)*, was not identified during the initial analysis for MA2003 because of inaccurate annotation at the splice acceptor site. Re-analysis identified this pathogenic variant as a result of improvements in determining the predicted loss of function variant.

## Discussion

Our independent re-analysis of exome data increased the diagnostic yield in both rare disease cases and sudden death by a combined rate of ~ 10%, consistent with the increased yield reported in prior studies [7–15]. Although any gain in diagnostic yield is of tremendous importance to those families receiving updated results, most of our cases remain unexplained after our re-analysis. It is possible that, given no new sequence, data was generated in this re-analysis that some portion of negative cases may be due to exomic variants not captured by our sequencing due to lack of coverage and/or improvements in sequencing chemistry over time. Other explanations include the inability to catalog all functional variants, especially non-coding regulatory and deep intronic variants, undiscovered gene-disease and/or gene-phenotype associations, the possibility of complicated oligogenic disease that is not easily dissected in small families, and the possibility of disease due to epigenetic, somatic, or other uninterrogated genomic aberrations. Further detection and interpretation of complex repeat expansions, copy-number variants, and structural variations could improve the diagnostic yield as it has been reported elsewhere though a direct interrogation of these structural variants outside of exome sequencing is preferred [36, 37].

The rapid pace at which novel disease genes and variants are discovered and reported as well as the continuous revision of genome annotation and the presence of new tools and genetic databases suggests that periodic re-analysis of undiagnosed WES participants should be actively performed. A plethora of additional candidate variants are uncovered as new evidence regarding gene-disease relationships and variant classifications comes to light, suggesting that automated methods for re-analysis which capture and evaluate the phenotypic correspondence between candidate variants and the observed phenotype are necessary to make this process efficient. While the absolute number of novel findings in our study is small, the 4 additional positive findings represent a substantial increase in relative diagnostic yield (18%). This increase in yield underscores the need for periodic re-interpretation and re-analysis of negative WES data for both rare disease and sudden death, particularly those cases not recently evaluated. Our novel findings were identified in cases 2+ years old. We found that no single factor was responsible for new findings but that updated annotations of gene models, variant pathogenicity, and gene-disease relationships automatically made and applied to WES cases can reveal a significant number of new diagnostic genetic variants. We suggest that a 6-month cycle of automated re-analysis could improve the pace at which new findings are disseminated to patients. Periodic re-analysis by third party or other software not originally used to analyze cases is also potentially useful to uncover pathogenic variants that may be missed by the differences across genome interpretation platforms.

## Conclusions

Continuous development in bioinformatics tool to classify and interpret variants, expansion of substantial exome resources, and advances in genomic knowledge highlight the critical need to revisit unsolved exome cases. Here we have demonstrated using an artificial intelligence-based variant classification and interpretation engine (Genoox; https://www.genoox.com) that re-evaluation of our exome cases increased the combined diagnostic yield by 10%. This result illustrates that periodic re-analysis of exome cases could reveal new diagnoses and give greater context for variant of uncertain significance. The identification of previously undetected diagnostic variants was the result of updated patient phenotype information, improved bioinformatics pipelines, and optimized variant interpretation workflow. Another potential source to enhance diagnostic yield could be attained through detection and characterization of structural genomic variants.

## Supplementary information

**Additional file 1: Table S1.** Number of classified variants in the Idiopathic Diseases of Human (IDIOM) study per participant. **Table S2.** Number of classified variants in the Molecular Autopsy (MA) study per participant. **Table S3.** A. Summary of the demographic characteristics and findings after re-analysis in the 51 cases of rare diseases from the Idiopathic Diseases of Human (IDIOM) study. **Table S3.** B. Summary of the demographic characteristics and autopsy findings after re-analysis in the 50 cases of sudden death in the young from the Molecular Autopsy (MA) study. **Table S4.** A. Annotation and classification of variants after re-analysis in the 51 cases of rare diseases from the Idiopathic Diseases in Human (IDIOM) study. **Table S4.** B. Annotation and classification of variants after re-analysis in the 50 cases of sudden death in the young from the Molecular Autopsy (MA) study.

## Data Availability

The datasets supporting the conclusions of this article are included within the article and its additional files. Due to patient privacy and data sharing consent, our raw data cannot be submitted to publicly available databases.

## Abbreviations

WES: Whole-exome sequencing
VUDS: Variant of uncertain diagnostic significance
PDV: Possible diagnostic variant
DV: Diagnostic variant
MA: Molecular Autopsy
IDIOM: Idiopathic Diseases Of huMan
GTR: Genetic Testing Registry
ACMG: American College of Medical Genetics and Genomics
AMP: Association for Molecular Pathology
